# Systematic review of risk assessment tools for post-discharge mortality among children in sub-Saharan Africa

**DOI:** 10.1371/journal.pgph.0004788

**Published:** 2025-07-01

**Authors:** Ramya Ginjupalli, Kaitlin Cole, Karim P. Manji, Rodrick Kisenge, Hannah Rogers, Adrianna Westbrook, Quique Bassat, Rosauro Varo, Lola Madrid, Inacio Mandomando, Claudia R. Morris, Nega Assefa, Richard Omore, Victor Akelo, Kitiezo Aggrey Igunza, Christopher P. Duggan, Chris A. Rees

**Affiliations:** 1 Emory University School of Medicine, Atlanta, Georgia, United States of America; 2 Department of Pediatrics and Child Health, Muhimbili University of Health and Allied Sciences, Dar es Salaam, Tanzania; 3 Woodruff Health Sciences Center Library, Emory University, Atlanta, Georgia, United States of America; 4 Pediatric Biostatistics Core, Department of Pediatrics, Emory University, Atlanta, Georgia, United States of America; 5 ISGlobal, Barcelona, Spain; 6 Centro de Investigação em Saúde de Manhiça [CISM], Maputo, Mozambique; 7 ICREA, Pg. Lluís Companys 23, Barcelona, Spain; 8 Institut Clínic de Medicina I Dermatologia, Hospital Clínic de Barcelona, Barcelona, Spain; 9 Facultat de Medicina i Ciències de la Salut, Universitat de Barcelona (UB), Barcelona, Spain; 10 Pediatrics Department, Hospital Sant Joan de Déu, Universitat de Barcelona, Esplugues, Barcelona, Spain; 11 CIBER de Epidemiología y Salud Pública, Instituto de Salud Carlos III, Madrid, Spain; 12 London School of Hygiene and Tropical Medicine, Keppel Street, London, United Kingdom; 13 College of Health and Medical Sciences, Harar, Haramaya University, Ethiopia; 14 Division of Pediatric Emergency Medicine, Emory University School of Medicine, Atlanta, Georgia, United States of America; 15 Department of Emergency Medicine, Children’s Healthcare of Atlanta, Atlanta, Georgia, United States of America; 16 Kenya Medical Research Institute-Center for Global Health Research, Kisumu, Kenya; 17 Liverpool School of Tropical Medicine, United Kingdom, Kenya; 18 Departments of Nutrition and Global Health and Population, Harvard T.H. Chan School of Public Health, Boston, Massachusetts, United States of America; 19 Center for Nutrition, Division of Gastroenterology, Hepatology, and Nutrition, Boston Children’s Hospital, Boston, Massachusetts, United States of America; Wageningen University & Research, NETHERLANDSKINGDOM OF THE

## Abstract

Post-discharge mortality is increasingly recognized as a major contributor to the high burden of childhood mortality in sub-Saharan Africa. Accurate identification of children at risk for post-discharge mortality is critically important to inform interventions to reduce deaths following hospital discharge. Our objective was to describe the current state of development, validation, or implementation for risk assessment tools for post-hospital discharge mortality (PDM) in sub-Saharan Africa. We conducted a systematic review of publications on risk assessment tools for PDM among children aged 0–18 years in sub-Saharan Africa. We searched CABI Global Health, Cochrane Reviews, Cochrane Trials, ProQuest Dissertations and Theses, Embase, PubMed, and Web of Science with no date or language restriction. We included publications if they described a tool/model with weights assigned to variables to quantify risk of PDM, included children, and were conducted in sub-Saharan Africa. We determined the level of evidence for tools using the Evidence-Based-Medicine Working Group hierarchy. Of 4,893 publications screened, 289 full texts were reviewed, and seven publications that reported 23 risk assessment tools for PDM among children in sub-Saharan Africa were identified. These studies enrolled 49,669 total participants (3.6%, n = 1,795 experienced PDM). There was substantial heterogeneity in identified risk factors, although all identified malnutrition as a risk factor for PDM. All risk assessment tools had fair (i.e., area under the receiver operating characteristic curve [AUC] ≥0.70) or good (AUC ≥ 0.80) discriminatory value in internal validation. Only two risk assessment tools had been externally validated, and none were implemented. Existing risk assessment tools to identify children at risk for PDM in sub-Saharan Africa lack broad validation and implementation. Malnutrition is a common risk factor for PDM. Further studies are needed to validate and implement such tools to reduce PDM among children.

## Introduction

Post-hospital discharge mortality (PDM), which include deaths that occur weeks to up to 12 months after hospital discharge, is increasingly recognized as a significant contributor to the high burden of childhood mortality in sub-Saharan Africa [1]. Recent studies suggest that as many as 3–13% of children who were hospitalized died within months of hospital discharge [2–4]. Prior systematic reviews and meta-analyses on PDM have focused on its overall burden and described individual risk factors associated with PDM [2,3,5]. However, prior reviews have not investigated approaches to accurately identify children at risk for PDM, which is crucial to tailoring interventions to reduce PDM.

Risk assessment tools, or clinical prediction scores, are powerful tools that use empiric clinical data to combine risk factors and assign numeric scores to variables to predict outcomes in situations of clinical uncertainty [6]. These tools have demonstrated efficacy in improving medical decision making, including in settings with limited resources [7,8]. In order for risk assessment tools to be used in clinical practice, they must first be rigorously derived, then internally validated to assess their ability to identify patients who may be at risk for a given outcome [9]. After internal validation, risk assessment tools should be externally validated and implemented to measure their impact on clinical outcomes.

To date, there have been no systematic reviews focused on risk assessment tools to identify children at risk for PDM. An understanding of the current state of risk assessment tool development for PDM may inform research and implementation gaps that need to be addressed to reduce the burden of PDM among children in sub-Saharan Africa. To this end, our objective was to review the current state of risk assessment tools derivation, validation, and implementation for PDM among children in sub-Saharan Africa.

## Materials and methods

### Study design

We conducted a systematic review of publications reporting risk assessment tools for PDM among children in sub-Saharan Africa. Risk assessment tools were defined as research-based tools that quantified the contributions of relevant patient characteristics to provide numeric indices that assist clinicians in making predictions regarding the presence, or the absence, of a condition, including variables at the time of, during, and following admission [6]. We followed the standards of the Preferred Reporting Items for Systematic Reviews and Meta-Analyses (PRISMA) statement [10]. Our study protocol was registered *a priori* in PROSPERO (CRD42024577326) [11]. As we used publicly available data, the study was deemed non-human research and did not require ethical approval.

### Search strategy

We identified publications on risk assessment tools to identify children (i.e., those aged 0–18 years at the time of hospitalization) at risk for PDM from any cause in sub-Saharan Africa. Because studies had different periods of follow up after hospitalization [3,12,13], we had no restriction on the follow-up period after hospital discharge for included publications. To reduce the likelihood of selection bias, we searched multiple publication databases including CABI Global Health, Cochrane Reviews, Cochrane Trials, ProQuest Dissertations and Theses, Embase (Elsevier), PubMed, and Web of Science. The search was conducted on July 30^th^, 2024, and encompassed all publications from the inception of each database to the search date. We also hand-searched key publications and systematic reviews related to PDM to ensure that no publications on risk assessment tools were excluded. We did not limit to any article type, publication date, or language in which the article was published. We developed our search strategy (S1 Appendix) in consultation with a librarian (HR) experienced in systematic reviews.

### Inclusion and exclusion criteria

We included studies if they met the following criteria (1) described a tool or model with weights assigned to variables to quantify risk of PDM, (2) were peer-reviewed publications (determined through journal name and review of accompanying author guidelines), (3) included children aged 0–18 years, and (4) took place, at least in part, in sub-Saharan Africa. As some studies included only specific patient populations (e.g., those with severe infections) [3], we included publications regardless of the specific population included. We used the World Bank’s list to identify sub-Saharan African countries [14]. We included publications on risk assessment tools that were derived in sub-Saharan Africa because this region had the highest reported rates of PDM at the time of our search [3,5]. We excluded publications if they occurred exclusively outside sub-Saharan Africa or if they reported an intervention for PDM without the inclusion of an approach to risk stratification.

### Selection process

Records from the initial search were downloaded using EndNote and uploaded to Covidence. Each record was reviewed independently by two of three authors (CAR, KC, and RG) to determine if they met inclusion criteria based on the title and abstract. During the screening stage, publications thought to not meet inclusion criteria by two authors were excluded. Publications in which there was disagreement between two reviewers were discussed and a third author assessed them for potential inclusion without knowledge of the previous reviewers’ recommendations. Publications that passed the title and abstract review phase underwent a full-text review for inclusion. As we aimed to determine the current state of risk assessment tool derivation, validation, and implementation [9], we searched for publications that had cited publications that reported the identified risk assessment tools for potential subsequent validation studies. Furthermore, we emailed the corresponding author of each identified publication to inquire if publications of studies on external validation of their tools were available. External validation was defined as the performance of the identified risk assessment tool in a cohort different from that used to create the risk assessment tool.

### Data extraction

Each included publication was read in detail independently by two reviewers and a structured data extraction tool (in Microsoft Excel) was used to extract the following pre-determined variables: study country or countries and site(s), age groups included, sample size, number of participants who had the outcome of PDM, study design, duration of study including follow-up period, patient characteristics used in risk assessment tool derivation, measured outcome, hazard or odds ratio assigned to each candidate variable, validation of the risk assessment tool, and discrimination of score measured by summary c-statistic or area under the receiver operating characteristic curve (AUC). Data were extracted by authors CAR and RG throughout October 2024 and re-confirmed on November 11^th^, 2024. All discrepancies in extracted data were discussed and publications were re-read until consensus was achieved. In cases where data were missing, the study authors were contacted for confirmation and the missing data was listed as “not reported”.

### Assessment of hierarchy of risk assessment tool validation

For each published risk assessment tool, we assessed the state of validation and implementation based on the Evidence-Based Medicine Working Group recommendations [15]. This hierarchy states that risk assessment tools meet one of four levels of evidence. Level 4 (lowest level) is assigned to risk assessment tools that have been derived but not externally validated. Level 3 risk assessment tools are those that have been externally validated in one sample. Risk assessment tools are assigned level 2 evidence when they have been broadly validated in multiple settings, and level 1 (highest level) risk assessment tools are those that have been implemented and impact analysis has been conducted. We also assessed whether risk assessment tools had been validated either temporally (i.e., validation done after derivation in the same setting) or geographically (i.e., validation conducted in a setting different from the derivation setting) [16].

### Risk of bias assessment

We used the Prediction Model Risk of Bias Assessment Tool (PROBAST) [17], a tool designed for bias assessment in prognostic predictive models, to assess bias in each publication. This assessment was completed independently by two authors (CAR, RG) and was reviewed for disagreement. Disagreements were discussed until consensus was achieved among the reviewers.

### Synthesis of evidence

A formal meta-analysis and pooled analysis was not possible given heterogeneity of both predictive variables included in the risk assessment tools and in reporting of test characteristics. We presented the main models from each publication in our main text and ancillary models are reported in the Supplement (S1 Table). Based on previously used standards, the discriminatory value of AUCs were classified as excellent (i.e., AUC ≥ 0.90), good (i.e., AUC 0.80 to 0.89), fair (i.e., AUC 0.70 to 0.79), and poor (i.e., AUC < 0.70) [8,18,19].

## Results

**Fig 1:**
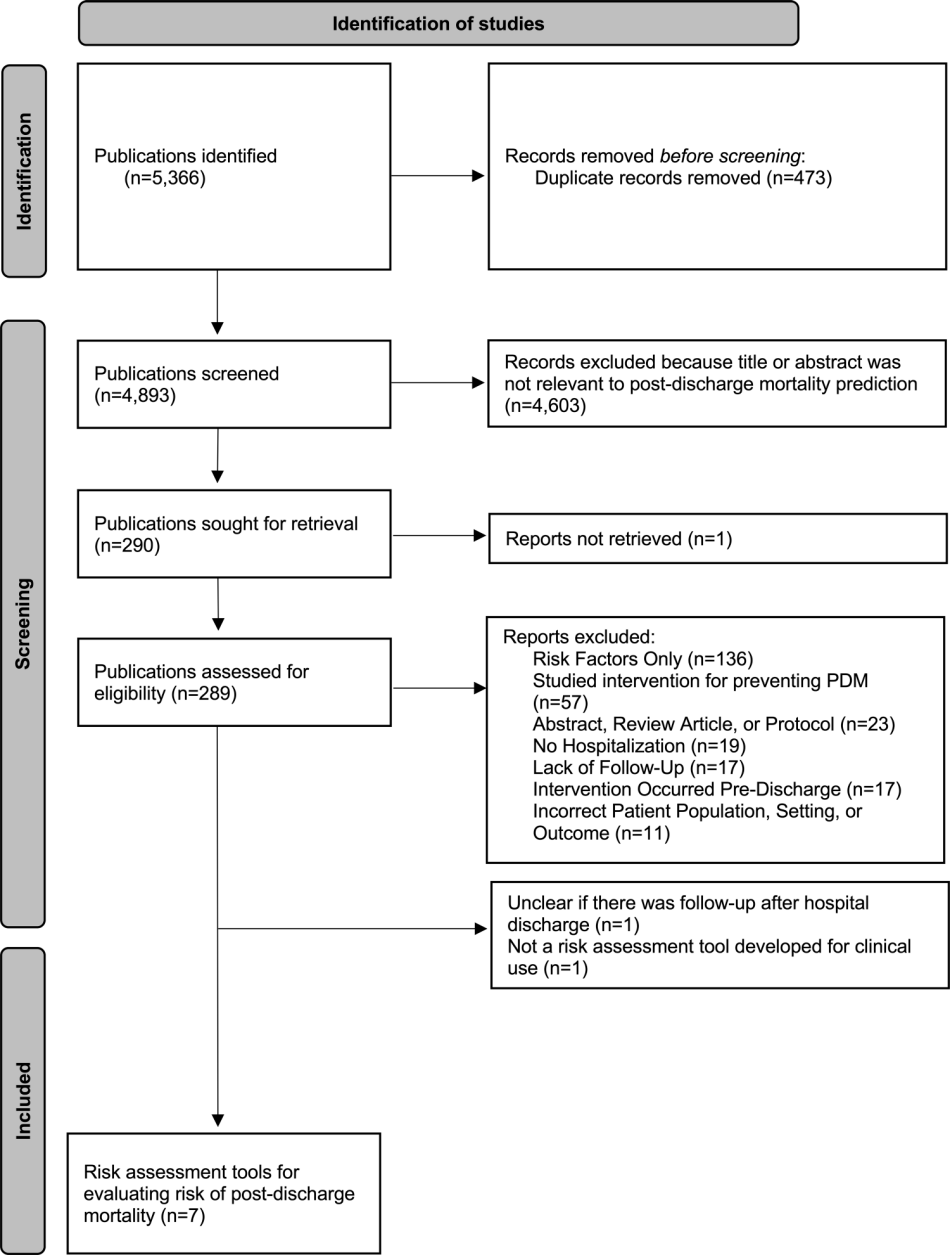
PRISMA diagram of included publications on risk assessment tools for post-discharge mortality among children in sub-Saharan Africa.

Our search resulted in 4,893 unique publications. Of these, 289 full texts were reviewed for eligibility and nine publications reported risk assessment tools for PDM among children in sub-Saharan Africa (Fig 1 and S2 Appendix). One study was later excluded as post-discharge follow-up could not be confirmed [20] and another was excluded because it provided an overview of a machine learning approach to identify risk factors for PDM, but did not include a risk assessment tool meant for clinical use [21]. Thus, seven publications were included in our report (Table 1) [22–28]. These seven publications included a total of 23 different risk assessment tools. These tools included combinations of demographic, socioeconomic, clinical signs, anthropometric measures, laboratory values (e.g., hemoglobin, blood culture positivity), and diagnoses obtained both at admission and at discharge.

**Table 1:** Characteristics of included publications on the derivation, validation, and implementation of risk assessment tools for post-discharge mortality among children in sub-Saharan Africa.

Reference	Study Design	Setting	Age Population	Hospital Diagnoses	Time of Variable Assessment	Population, n	Post-Discharge Mortality Cases, n (% of overall population)	Outcome and Follow-Up Timeframe
Talbert A, et al. *BMC Med*. 2019.	Retrospective cohort	One rural hospital in Kenya	2-59 months	Diarrheal disease	Hospital admission	2,394	49 (1.9)	All-cause mortality ≤1 year after discharge
Ahmed SM, et al. *PLOS Glob Public Health*. 2023.	Prospective case control	Seven urban and rural hospitals in The Gambia, Mali, Mozambique, Kenya, India, Bangladesh, and Pakistan	0-59 months	Moderate to severe diarrheal disease	Hospital admission	8,017	122 (1.5)	All-cause mortality ≤91 days of treatment
Wiens MO, et al. *BMJ Open*. 2015.	Prospective cohort	Two urban hospitals in Uganda	6-60 months	Suspected or proven infection	Hospital admission and discharge	1,242	61 (4.9)	All-cause mortality ≤180 days of discharge
Wiens MO, et al. *PLOS Glob Public Health*. 2024.	Prospective cohort	Six urban and rural hospitals in Uganda	0-6 months	Suspected or proven infection	Hospital admission and discharge	3,349	257 (7.7)	All-cause mortality ≤180 days of discharge
			6-60 months	Suspected or proven infection	Hospital admission and discharge	4,830	233 (4.8)	All-cause mortality ≤180 days of discharge
Madrid L, et al. *Pediatrics*. 2019.	Retrospective cohort	One district hospital in Mozambique	0-14 years	Any diagnosis	Hospital admission and discharge	18,023	935 (3.6)	All-cause mortality ≤90 days after discharge
Rees CA, et al. *J Pediatrics*. 2024.	Prospective cohort	Two urban referral hospitals in Tanzania and Liberia	1-59 months	Any diagnosis	Hospital admission and discharge	1,933	67 (3.5)	All-cause mortality ≤60 days after discharge
Rees CA, et al. *BMJ Open*. 2024.	Prospective cohort	Two urban referral hospitals in Tanzania and Liberia	0-28 days	Any diagnosis	Hospital admission and discharge	2,310	71 (3.1)	All-cause mortality ≤60 days after discharge

The total number of participants enrolled in the included studies was 49,669, of whom 3.6% (n = 1,795) experienced PDM. Participants were observed following discharge anywhere from 60 days up to 365 days; three of the included publications described 180-day follow-up. The identified tools were derived in Uganda, Mozambique, Kenya, Tanzania, Liberia, The Gambia, and Mali. In addition to sites in sub-Saharan Africa, one study included study sites in Pakistan and India (Table 1) [25]. Four of the seven included studies were conducted in single countries.

The age of included participants ranged from 0 days to 14 years, and the time after discharge of deaths ranged between 60 days and 12 months. One study enrolled neonates aged 0–28 days at the time of discharge [27], and one study included participants aged 1 day up to 14 years of age [23]. Two of the studies included children admitted with diarrheal disease [24,25], two included children with suspected or proven infections [22,28], and three included children admitted with any diagnosis [23,26,27].

### Risk assessment tools for PDM for children admitted with diarrheal disease

Two studies developed risk assessment tools for PDM for children admitted with diarrhea (Table 1). One of these was a retrospective cohort study in Kenya with 2,394 participants aged 2–59 months admitted with diarrhea conducted in a county hospital and followed patients up to 365 days after discharge [24]. These investigators used logistic regression to develop a risk assessment tool from 24 candidate variables. They developed a model that included 5 variables assigned weights with hazard ratios (Table 2). The most predictive factors for PDM were HIV infection, low mid-upper arm circumference (MUAC), bacteremia, prior hospital admissions, and chest wall indrawing. In internal validation, this tool demonstrated good discriminatory value (AUC 0.87).

**Table 2:** Risk factors and discriminatory value of main risk assessment tools for post-discharge mortality among children in sub-Saharan Africa.

Reference	Risk Factors	Hazard Ratio* or Odds Ratio** (95% Confidence Interval [CI])	Area Under Receiver Operating Characteristic Curve (95% CI) in Internal Validation	Area Under Receiver Operating Characteristic Curve (95% CI) in External Validation
*Populations with Hospital Diagnoses of Diarrhea*				
Talbert A, et al. *BMC Med*. 2019.	Prior hospital admission	3.11* (1.57 to 6.17)	0.87 (0.81 to 0.94)	No Published External Validation Studies
	Lower chest wall indrawing	2.00 (1.03 to 3.79)		
	HIV antibody positive	5.02 (2.31 to 10.92)		
	Bacteremia	3.69 (1.64 to 10.14)		
	Mid-upper arm circumference (MUAC) (per centimeter)	0.67 (0.56 to 0.81)		
Ahmed SM, et al. *PLOS Glob Public Health*. 2023.^^^	MUAC	0.48** (0.43 to 0.54)	0.85 (0.83 to 0.87)	0.74 (0.71 to 0.77)
	Respiratory rate	1.03 (1.01 to 1.04)		
	Temperature	1.51 (1.28 to 1.78)		
	Age in months	1.02 (1.00 to 1.03)		
	n people living in household	1.00 (0.97 to 1.02)		
	n days of diarrhea at presentation	1.07 (0.95 to 1.21)		
	Since diarrhea starts, how much offering child to drink	1.35 (1.16 to 1.57)		
	n children aged <60 months in household	0.98 (0.88 to 1.09)		
	Abnormal hair (sparse, loose, straight)	4.03 (2.61 to 6.14)		
	n rooms in house used for sleeping	1.02 (0.96 to 1.09)		
*Populations with Suspected or Proven Infections During Hospitalization*				
Wiens MO, et al. *BMJ Open*. 2015. (Model 1)	MUAC	0.95** (0.94 to 0.97)	0.82 (0.75 to 0.87)	No Published External Validation Studies
	SpO_2_	0.96 (0.93 to 0.99)		
	Time since last hospitalization	0.76 (0.62 to 0.93)		
	HIV positive	2.98 (1.36 to 6.53)		
	Abnormal BCS score	2.39 (1.18 to 4.83)		
Wiens MO, et al. *PLOS Glob Public Health*. 2024. (Aged 0–6 months, M6PD-A_0-6_)	Age	Not reported	0.77 (not reported)	0.71 (not reported)
	Duration of present illness			
	MUAC			
	Neonatal jaundice			
	Sucking well when breastfeeding			
	SpO_2_			
	Time to reach hospital			
	Weight for age z-score			
Wiens MO, et al. *PLOS Glob Public Health*. 2024. (Aged 6–60 months, M6PD-A_6-60_)	Age	Not reported	0.77 (not reported)	0.74 (not reported)
	Hemoglobin			
	HIV			
	How long since last admission			
	MUAC			
	SpO_2_			
	Water source			
	Weight for age z-score			
*Populations Admitted with All Diagnoses*				
Madrid L, et al. *Pediatrics*. 2019. (Model 1, 90-day outcome)	Age 4m- < 1 y	0.92 (0.71 to 1.20)	0.83 (0.81 to 0.84)	No Published External Validation Studies
	Age 1–5 y	0.69 (0.53 to 0.91)		
	Age > 5 y	0.54 (0.38 to 0.76)		
	Rainy season	1.22 (1.03 to 1.43)		
	WHZ z score >−2 to <−1	1.23 (0.75 to 2.01)		
	WHZ z score >−3 to <−2	2.40 (1.49 to 3.87)		
	WHZ z score <−3	3.26 (2.08 to 5.12)		
	WHZ z score Unknown	2.99 (2.12 to 4.21)		
	Diarrhea	1.72 (1.45 to 2.03)		
	Cough	1.32 (1.07 to 1.62)		
	Increased respiratory rate	1.41 (1.18 to 1.68)		
	Nasal flaring	0.69 (0.55 to 0.86)		
	Auscultatory crackles	1.37 (1.12 to 1.67)		
	Oral candidiasis	2.64 (1.98 to 3.52)		
	Edema	1.86 (1.39 to 2.48)		
	Depigmented/reddish hair	2.03 (1.60 to 2.57)		
	Swollen lymph nodes	1.89 (1.42 to 2.51)		
	Ear discharge	1.76 (1.20 to 2.58)		
	Prostration	1.42 (1.15 to 1.75)		
	Malaria Positive	0.44 (0.36 to 0.54)		
	Malaria Test not done	0.86 (0.46 to 0.73)		
	Blood Culture Positive	1.68 (1.33 to 2.12)		
	HIV Positive	1.77 (1.07 to 2.91)		
	HIV Negative	0.53 (0.35 to 0.80)		
	Outcome of Admission: Absconded	5.23 (4.22 to 6.50)		
	Outcome of Admission: Transferred	4.48 (3.31 to 6.05)		
Rees CA, et al. *J Pediatr*. 2024.	Left against medical advice	6.46** (2.46 to 15.3)	0.77 (0.76 to 0.78)	No Published External Validation Studies
	Male	1.86 (1.06 to 3.38)		
	Pedal edema anytime during hospital admission	6.94 (1.69 to 22.6)		
	Cyanosis during hospital admission	4.55 (1.59 to 11.4)		
	Pallor during hospital admission	1.98 (1.03 to 3.63)		
	MUAC <11.5 cm	3.55 (1.96 to 6.33)		
	Cancer	10.6 (2.58 to 34.6)		
	HIV	2.74 (0.52 to 9.91)		
	Seizures	0.16 (0.01 to 1.22)		
	Presence of any chronic medical conditions	2.51 (1.35 to 4.49)		
*Neonatal Population Admitted with All Diagnoses*				
Rees CA, et al. *BMJ Open*. 2024.	Left against medical advice	5.62** (2.40 to 12.1)	0.77 (0.75 to 0.80)	No Published External Validation Studies
	Mother had ≥ 3 prior deliveries	1.83 (1.09 to 3.05)		
	Family home is near a hospital	0.33 (0.06 to 1.04)		
	Pallor observed by clinician	3.79 (1.32 to 9.43)		
	Low birth weight (i.e., < 2500 g)	3.14 (1.83 to 5.44)		
	Discharge temperature 35.5–37.9ºC	0.2 (0.08 to 0.59)		
	Received supplemental oxygen during hospital admission	1.86 (1.07 to 3.15)		
	Meconium aspiration pneumonia	6.98 (1.69 to 21.7)		
	Congenital birth defects	4.55 (1.70 to 10.6)		
	Hematological diseases	3.64 (0.72 to 13.2)		

^ Ahmed et al. reported odds ratios for their variables for death for both inpatient and post-discharge deaths, though the test characteristics are for post-discharge mortality.

The other risk assessment tool was developed by investigators from the Global Enteric Multicenter Study (GEMS), which was a prospective case control study with 8,017 participants aged 0–59 months presenting with diarrhea conducted in Mali, The Gambia, Kenya, Mozambique, Bangladesh, India, and Pakistan followed 91 days after discharge [25]. These investigators used logistic regression to develop a risk assessment tool from 130 candidate variables to identify children at risk for PDM from all sites except for Kenya (i.e., the geographical validation cohort). Their final tool included 10 variables that were assigned weights reported as odds ratios (Table 2). MUAC at presentation, temperature, oral hydration since the onset of the diarrheal illness, and abnormal hair (sparse, loose, straight) were most predictive of PDM. In internal validation, this tool had good discriminatory value (AUC 0.86) and fair discriminatory value (AUC 0.74) in external validation at the GEMS study site in Kenya.

### Risk assessment tools for PDM for children admitted with suspected or proven infections

Two studies developed risk assessment tools for PDM 180 days after discharge in children who were admitted to eight hospitals with the diagnosis of “suspected or proven infection”. A prospective cohort study in Uganda with 1,242 participants aged 6–60 months admitted to two hospitals followed patients up to 180 days after discharge [22]. These investigators used logistic regression to develop four risk assessment tools from 36 candidate variables (Table 2). Their tools included three to five variables (Table 2 and S1 Table). Factors most predictive of PDM were MUAC, time since last hospitalization, oxygen saturation, abnormal Blantyre Coma Score, and HIV-positive status. In internal validation, these tools had good discriminatory value (AUC 0.80-82).

A prospective cohort study in Uganda with 8,179 participants aged 0–60 months admitted with suspected sepsis conducted in six hospitals followed patients up to 180 days [28]. Using logistic regression, these investigators developed six risk assessment tools (i.e., 3 for infants aged 0–6 months and 3 for children aged 6–60 months) from 80 candidate variables (Table 2 and S1 Table). The most predictive factors were anthropometry, illness duration, jaundice-age interaction, prior admissions, temperature, age-respiratory rate interaction, and HIV status. In internal validation, their tools had fair discriminatory value (AUC 0.75-0.77) and in external validation in a cohort study conducted at two hospitals in Rwanda, three of the tools had fair discriminatory value (AUC 0.71-0.75) [29].

### Risk assessment tools for PDM for children admitted with all diagnoses

Two studies identified and quantified risk factors associated with PDM. A retrospective cohort study in Mozambique with 18,023 participants aged <15 years conducted in a district hospital followed patients up to 90 days after discharge [23]. These investigators used logistic regression to develop nine risk assessment tools from 45 candidate variables. Their analysis resulted in tools with seven variables to identify children at risk for 30-, 60-, or 90-day PDM. The most predictive factors were young age, moderate or severe malnutrition, a history of diarrhea, HIV infection, and discharge against medical advice. Their tools had fair to good discriminatory value in internal validation (AUC 0.75-0.81).

A prospective cohort study in Tanzania and Liberia with 1,933 participants aged 1–59 months conducted in two hospitals followed patients up to 60 days after discharge [26]. These investigators used logistic regression to develop a risk assessment tool from 121 candidate variables. Their analysis resulted in a tool including 10 variables that were assigned weighted points (Table 2). The most predictive factors for PDM were leaving against medical advice, pedal edema during hospital admission, cyanosis during hospital admission, MUAC <11.5 cm, and cancer diagnosis. In internal validation, this tool had an AUC of 0.77 (i.e., fair discriminatory value).

### Risk assessment tool for PDM for admitted neonates

A prospective cohort study in Tanzania and Liberia enrolled 2,310 participants aged 0–28 days conducted in two hospitals followed patients up to 60 days after discharge [27]. These investigators used logistic regression to develop a risk assessment tool from 115 candidate variables. Their analysis resulted in a 10-variable tool with variables assigned weights in the form of points (Table 2). The most predictive factors for PDM were leaving against medical advice, diagnosis of meconium aspiration, pallor observed by clinician, low birth weight, congenital birth defects, and hematological diseases (i.e., anemia, hemoglobinopathies, and vitamin K deficiency in the newborn). In internal validation, this tool demonstrated fair discriminatory value (AUC 0.77).

### Assessment of risk of bias and hierarchy of risk assessment tool validation

**Fig 2:**
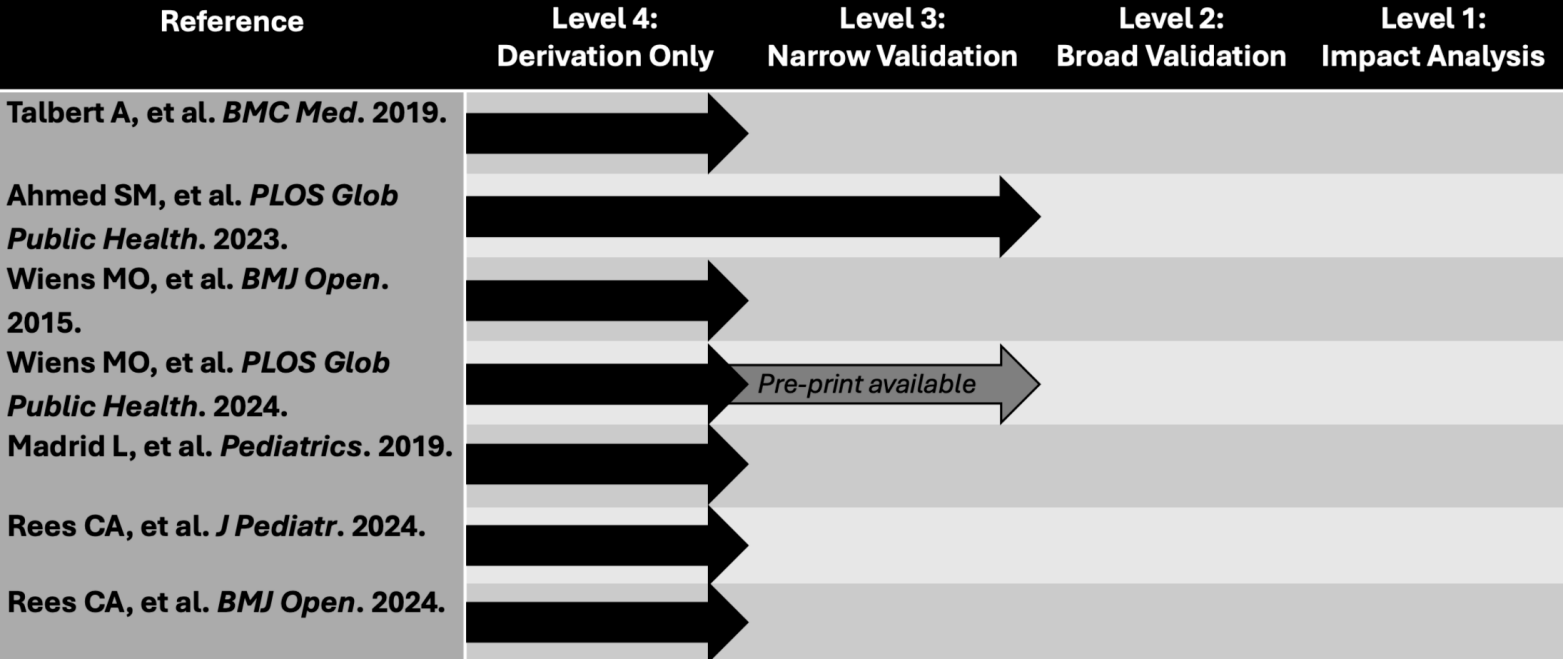
Current state of derivation, validation, and implementation of risk assessment tools for post-discharge mortality among children in sub-Saharan Africa based on the Evidence-Based Medicine Working Group hierarchy of evidence.

One publication was low risk for bias and six publications had unclear risk for bias because at least one element was unclear per PROBAST (S2 Table). Of the seven included publications of risk assessment tools, six studies developed and internally validated a tool and one study developed and externally validated their tool in the same publication for the prediction of PDM among children with diarrheal disease (Fig 2). From our inquiries to corresponding authors, we identified a pre-print of an external validation study of the tools originally developed in Uganda for children with suspected sepsis (obtained from Wiens MO on December 10, 2024) [29]. None of the tools had published reports of their implementation and their impact on PDM. However, there was a continuation study conducted in the same facilities where the risk assessment tool by Wiens MO, et al. was developed that did not use this tool for risk differentiation for the intervention of a bundle of interventions that was given to all discharged patients [30].

## Discussion

Our systematic review identified seven publications that reported 23 risk assessment tools to identify children at risk for PDM in sub-Saharan Africa. These publications reported >60 unique risk factors predictive of PDM. The studies were conducted across 10 countries and included populations varying in age and diagnoses. Despite such heterogeneity, all risk assessment tools included malnutrition (or a clinical indicator of it) as an independent predictive factor for PDM. All identified risk assessment tools had fair or good discriminatory value in internal validation. However, only one of the identified risk assessment tools had a published report of external validation and one had a reported external validation that appeared in a pre-print. None of the risk assessment tools had reports of their implementation or their impact on PDM reduction.

There was heterogeneity in included populations, study settings, and candidate risk factors in the included risk assessment tools for PDM among children in sub-Saharan Africa. Most identified risk factors can be categorized as social or environmental factors, which highlights the importance of identifying and addressing suboptimal socioeconomic factors when predicting PDM. Previous studies have shown place of residence, the child’s sex, mother’s educational level, and household wealth are predictors of under-five mortality in sub-Saharan Africa [31]. Four of the included tools identified that participants who were discharged against medical advice had greater risk of PDM. In a separate study [32], leaving against medical advice was shown to be associated with mortality after discharge regardless of initial exposure condition. This is a sociologic phenomenon that may be the result of financial constraints due to many factors such as the cost of ongoing clinical care or the cost of removing a dead body from the hospital. Moreover, perceived hopelessness from poor prognosis, or inadequate counseling by healthcare providers to families may also lead to discharge against medical advice [33].

Malnutrition was identified as a risk factor for PDM across all studies, though the way it was ultimately represented in the final risk assessment tools varied across studies, including MUAC, low birth weight, weight-for-age, and/or weight-for-length. This aligns with prior work that suggests that, in some countries in sub-Saharan Africa, malnutrition is a causal or significant condition in 40–45% of infant and child deaths [34,35]. It is vital for clinicians to recognize malnutrition as a strong predictive factor of PDM as such children may benefit from either delayed discharge, or nutritional programs following hospital discharge [36,37].

Only one of the risk assessment tools had a published report of external validation and one had a pre-print of an external validation study at the time of our review. Nonetheless, none of the identified tools had reports of implementation or impact analysis, which is how such tools ultimately may benefit patients. The development of risk assessment tools is an important first step in identifying patients at risk for PDM but should not be interpreted as a replacement for effective interventions, which have demonstrated mixed results in other studies [13,38,39]. However, prior intervention studies have not used precise approaches to patient identification as is allotted by risk assessment tools. Due to the varying epidemiology of diseases and resource availability across sub-Saharan Africa, accuracy of risk assessment tools may differ if the implemented region is substantially different from the region of development. Additionally, the accuracy of risk assessment tools for PDM may vary when applied to age groups or populations different from those in which the tool was developed.

Only two tools included numerical scores that can be utilized without the use of a calculator or a smartphone application [26,27]. In their current state, the other tools may be difficult for practitioners to use to quantify individual patient risk. Thus, risk assessment tools must be translatable at the bedside such that clinicians may use them in routine practice. Mobile health tools, such as the PAediatric Risk Assessment Mobile App, have been developed and implemented to identify such at-risk patients and improve post-discharge outcomes [40], but their impact on PDM has yet to be explored.

Certain predictors, such as MUAC and weight-for-age z scores, are commonly assessed at time of admission. However, measuring these metrics at discharge rather than admission may yield distinct predictive values for post-discharge outcomes. Thus, during implementation, anthropometry may be best measured at discharge to inform risk differentiated approaches for PDM. Furthermore, some of the features included in the risk assessment tools may not be routinely available in clinical practice in some settings in sub-Saharan Africa (e.g., bacteremia or cancer), which may limit their generalizability or, as diagnostic capacity expands in sub-Saharan Africa, may be more readily available as health systems are strengthened.

### Limitations

It is important to consider the limitations in our study. It is possible that some publications on the development of risk assessment tools for PDM were not found within our search parameters. We mitigated this risk through the search of several databases and a rigorous approach to publication review both at the screening and review phases. The identified risk assessment tools have not been compared head-to-head in the same populations, making it difficult to comment on the optimal tool to be targeted for future validation studies. Due to heterogeneity of populations and risk factors identified, a formal meta-analysis was not possible.

## Conclusions

The current body of research on risk assessment tools for predicting post-discharge mortality among children in sub-Saharan Africa includes various risk factors, including malnutrition which appeared in every identified study. However, many of the developed risk assessment tools for post-discharge mortality among children lack broad external validation and implementation. Future studies are urgently needed to externally validate existing risk assessment tools followed by implementation of these tools to aid in the reduction of post-discharge mortality among children in sub-Saharan Africa.

## Supporting information

S1 Table Additional reported risk factors and discriminatory value of models for post-discharge mortality among children in sub-Saharan Africa. (DOCX)

S2 Table Bias assessment for risk assessment tools for post-discharge mortality prediction among children in sub-Saharan Africa according to the Prediction Model Risk of Bias Assessment Tool (PROBAST). (DOCX)

S1 Appendix Search terms used to identify publications reporting on risk assessment tools for post-discharge mortality among children in sub-Saharan Africa. (DOCX)

S2 Appendix Full list of identified publications and reasons for inclusion or exclusion. (CSV)
