# Supplementary material for: Systematic review of risk assessment tools for post-discharge mortality among children in sub-Saharan Africa
Source: PLOS Glob Public Health. 2025 Jul 1;5(7):e0004788. doi: 10.1371/journal.pgph.0004788 (PMC12212496; doi:10.1371/journal.pgph.0004788)
Supplement: S1 Table — (DOCX) [file pgph.0004788.s001.docx]

**S1 Table.** Additional reported risk factors and discriminatory value of models for post-discharge mortality among children in sub-Saharan Africa

| **Reference** | **Risk Factors** | **Hazard Ratio* or Odds Ratio****  **(95% Confidence Interval [CI])** | **Area Under Receiver Operating Characteristic Curve (95% CI) in Internal Validation** | **Area Under Receiver Operating Characteristic Curve (95% CI) in External Validation** |
| --- | --- | --- | --- | --- |
| *Populations with Suspected or Proven Infections During Hospitalization* | | | | |
| Wiens MO, et al. *BMJ Open*. 2015. (Model 2) | MUAC | 0.95** (0.94 to 9.97) | 0.81 (0.75 to 0.87) | No Published External Validation Studies |
|  | Time since last hospitalization | 0.78 (0.64 to 0.95) |  |  |
|  | HIV positive | 2.98 (1.37 to 6.48) |  |  |
|  | Abnormal BCS score | 2.91 (1.47 to 5.75) |  |  |
|  | | | | |
| Wiens MO, et al. *BMJ Open*. 2015. (Model 3) | MUAC | 0.95** (0.94 to 0.97) | 0.80 (0.74 to 0.86) | No Published External Validation Studies |
|  | SpO2 | 0.96 (0.93 to 0.99) |  |  |
|  | Time since last hospitalization | 0.75 (0.61 to 0.92) |  |  |
|  | Abnormal BCS score | 2.23 (1.11 to 4.51) |  |  |
|  | | | | |
| Wiens MO, et al. *BMJ Open*. 2015. (Model 4) | MUAC | 0.95** (0.94 to 0.97) | 0.80 (0.73 to 0.86) | No Published External Validation Studies |
|  | Time since last hospitalization | 0.78 (0.64 to 0.96) |  |  |
|  | HIV positive | 2.76 (1.26 to 6.01) |  |  |
|  | | | | |
| Wiens MO, et al. *PLOS Glob Public Health*. 2024. (Aged 0-6 months, M6PD-C_0-6_) | Age | Not reported | 0.77 (not reported) | 0.75 (not reported) |
|  | Duration of present illness |  |  |  |
|  | MUAC |  |  |  |
|  | Neonatal jaundice |  |  |  |
|  | Sucking well when breastfeeding |  |  |  |
|  | SpO_2_ |  |  |  |
|  | Weight for age z-score |  |  |  |
|  | Fontanelle bulging |  |  |  |
|  | | | | |
| Wiens MO, et al. *PLOS Glob Public Health*. 2024. (Aged 0-6 months, M6PD-CS_0-6_) | Age | Not reported | 0.77 (not reported) | 0.71 (not reported) |
|  | Duration of present illness |  |  |  |
|  | MUAC |  |  |  |
|  | Neonatal jaundice |  |  |  |
|  | Sucking well when breastfeeding |  |  |  |
|  | SpO_2_ |  |  |  |
|  | Time to reach hospital |  |  |  |
|  | Weight for age z-score |  |  |  |
|  | | | |  |
| Wiens MO, et al. *PLOS Glob Public Health*. 2024. (Aged 6-60 months, M6PD-C_6-60_) | Age | Not reported | 0.75 (not reported) | No Published External Validation Studies |
|  | How long since last admission |  |  |  |
|  | MUAC |  |  |  |
|  | SpO_2_ |  |  |  |
|  | Weight for age z-score |  |  |  |
|  | Abnormal Blantyre coma scale |  |  |  |
|  | Respiratory rate |  |  |  |
|  | Temperature |  |  |  |
|  | | | | |
| Wiens MO, et al. *PLOS Glob Public Health*. 2024. (Aged 6-60 months, M6PD-CS_6-60_) | Age | Not reported | 0.76 (not reported) | 0.72 (not reported) |
|  | HIV |  |  |  |
|  | How long since last admission |  |  |  |
|  | MUAC |  |  |  |
|  | SpO_2_ |  |  |  |
|  | Water source |  |  |  |
|  | Weight for age z-score |  |  |  |
|  | Boil/disinfect/filter water |  |  |  |
| *Populations Admitted with All Diagnoses* | | | | |
| Madrid L, et al. *Pediatrics*. 2019. (Model 1, 30-day outcome) | Age 4m-<1 y | 0.92* (0.71 to 1.20) | 0.81 (0.76 to 0.86) | No Published External Validation Studies |
|  | Age 1-5 y | 0.69 (0.53 to 0.91) |  |  |
|  | Age >5 y | 0.54 (0.38 to 0.76) |  |  |
|  | Rainy season | 1.22 (1.03 to 1.43) |  |  |
|  | WHZ z score >−2 to <−1 | 1.23 (0.75 to 2.01) |  |  |
|  | WHZ z score >−3 to <−2 | 2.40 (1.49 to 3.87) |  |  |
|  | WHZ z score <−3 | 3.26 (2.08 to 5.12) |  |  |
|  | WHZ z score Unknown | 2.99 (2.12 to 4.21) |  |  |
|  | Diarrhea | 1.72 (1.45 to 2.03) |  |  |
|  | Cough | 1.32 (1.07 to 1.62) |  |  |
|  | Increased respiratory rate | 1.41 (1.18 to 1.68) |  |  |
|  | Nasal flaring | 0.69 (0.55 to 0.86) |  |  |
|  | Auscultatory crackles | 1.37 (1.12 to 1.67) |  |  |
|  | Oral candidiasis | 2.64 (1.98 to 3.52) |  |  |
|  | Edema | 1.86 (1.39 to 2.48) |  |  |
|  | Depigmented/reddish hair | 2.03 (1.60 to 2.57) |  |  |
|  | Swollen lymph nodes | 1.89 (1.42 to 2.51) |  |  |
|  | Ear discharge | 1.76 (1.20 to 2.58) |  |  |
|  | Prostration | 1.42 (1.15 to 1.75) |  |  |
|  | Malaria Positive | 0.44 (0.36 to 0.54) |  |  |
|  | Malaria Test not done | 0.86 (0.46 to 0.73) |  |  |
|  | Blood Culture Positive | 1.68 (1.33 to 2.12) |  |  |
|  | HIV Positive | 1.77 (1.07 to 2.91) |  |  |
|  | HIV Negative | 0.53 (0.35 to 0.80) |  |  |
|  | Outcome of Admission: Absconded | 5.23 (4.22 to 6.50) |  |  |
|  | Outcome of Admission: Transferred | 4.48 (3.31 to 6.05) |  |  |
|  | | | | |
| Madrid L, et al. *Pediatrics*. 2019. (Model 1, 60-day outcome) | Age 4m-<1 y | 0.92* (0.71 to 1.20) | 0.80 (0.76 to 0.84) | No Published External Validation Studies |
|  | Age 1-5 y | 0.69 (0.53 to 0.91) |  |  |
|  | Age >5 y | 0.54 (0.38 to 0.76) |  |  |
|  | Rainy season | 1.22 (1.03 to 1.43) |  |  |
|  | WHZ z score >−2 to <−1 | 1.23 (0.75 to 2.01) |  |  |
|  | WHZ z score >−3 to <−2 | 2.40 (1.49 to 3.87) |  |  |
|  | WHZ z score <−3 | 3.26 (2.08 to 5.12) |  |  |
|  | WHZ z score Unknown | 2.99 (2.12 to 4.21) |  |  |
|  | Diarrhea | 1.72 (1.45 to 2.03) |  |  |
|  | Cough | 1.32 (1.07 to 1.62) |  |  |
|  | Increased respiratory rate | 1.41 (1.18 to 1.68) |  |  |
|  | Nasal flaring | 0.69 (0.55 to 0.86) |  |  |
|  | Auscultatory crackles | 1.37 (1.12 to 1.67) |  |  |
|  | Oral candidiasis | 2.64 (1.98 to 3.52) |  |  |
|  | Edema | 1.86 (1.39 to 2.48) |  |  |
|  | Depigmented/reddish hair | 2.03 (1.60 to 2.57) |  |  |
|  | Swollen lymph nodes | 1.89 (1.42 to 2.51) |  |  |
|  | Ear discharge | 1.76 (1.20 to 2.58) |  |  |
|  | Prostration | 1.42 (1.15 to 1.75) |  |  |
|  | Malaria Positive | 0.44 (0.36 to 0.54) |  |  |
|  | Malaria Test not done | 0.86 (0.46 to 0.73) |  |  |
|  | Blood Culture Positive | 1.68 (1.33 to 2.12) |  |  |
|  | HIV Positive | 1.77 (1.07 to 2.91) |  |  |
|  | HIV Negative | 0.53 (0.35 to 0.80) |  |  |
|  | Outcome of Admission: Absconded | 5.23 (4.22 to 6.50) |  |  |
|  | Outcome of Admission: Transferred | 4.48 (3.31 to 6.05) |  |  |
|  | | | | |
| Madrid L, et al. *Pediatrics*. 2019. (Model 2, 30-day outcome) | Age 4m-<1 y | 0.93* (0.72 to 1.20) | 0.81 (0.76 to 0.86) | No Published External Validation Studies |
|  | Age 1-5 y | 0.71 (0.54 to 0.92) |  |  |
|  | Age >5 y | 0.54 (0.38 to 0.76) |  |  |
|  | Rainy season | 1.22 (1.04 to 1.44) |  |  |
|  | WHZ z score >−2 to <−1 | 1.27 (0.77 to 2.07) |  |  |
|  | WHZ z score >−3 to <−2 | 2.44 (1.51 to 3.93) |  |  |
|  | WHZ z score <−3 | 3.28 (2.08 to 5.16) |  |  |
|  | WHZ z score Unknown | 3.09 (2.19 to 4.35) |  |  |
|  | Diarrhea | 1.70 (1.44 to 2.01) |  |  |
|  | Cough | 1.31 (1.07 to 1.61) |  |  |
|  | Increased respiratory rate | 1.42 (1.19 to 1.69) |  |  |
|  | Nasal flaring | 0.69 (0.56 to 0.87) |  |  |
|  | Auscultatory crackles | 1.41 (1.16 to 1.71) |  |  |
|  | Oral candidiasis | 2.72 (2.03 to 3.64) |  |  |
|  | Edema | 1.83 (1.67 to 2.44) |  |  |
|  | Depigmented/reddish hair | 2.08 (1.64 to 2.64) |  |  |
|  | Swollen lymph nodes | 1.87 (1.41 to 2.49) |  |  |
|  | Ear discharge | 1.74 (0.59 to 1.16) |  |  |
|  | Prostration | 1.44 (1.17 to 1.77) |  |  |
|  | Malaria Positive | 0.43 (0.35 to 0.52) |  |  |
|  | Malaria Test not done | 0.84 (0.68 to 1.04) |  |  |
|  | HIV Positive | 1.80 (1.07 to 3.01) |  |  |
|  | HIV Negative | 0.53 (0.35 to 0.80) |  |  |
|  | Outcome of Admission: Absconded | 5.50 (4.45 to 6.79) |  |  |
|  | Outcome of Admission: Transferred | 4.57 (3.36 to 6.21) |  |  |
|  | | | | |
| Madrid L, et al. *Pediatrics*. 2019. (Model 2, 60-day outcome) | Age 4m-<1 y | 0.93* (0.72 to 1.20) | 0.80 (0.76 to 0.84) | No Published External Validation Studies |
|  | Age 1-5 y | 0.71 (0.54 to 0.92) |  |  |
|  | Age >5 y | 0.54 (0.38 to 0.76) |  |  |
|  | Rainy season | 1.22 (1.04 to 1.44) |  |  |
|  | WHZ z score >−2 to <−1 | 1.27 (0.77 to 2.07) |  |  |
|  | WHZ z score >−3 to <−2 | 2.44 (1.51 to 3.93) |  |  |
|  | WHZ z score <−3 | 3.28 (2.08 to 5.16) |  |  |
|  | WHZ z score Unknown | 3.09 (2.19 to 4.35) |  |  |
|  | Diarrhea | 1.70 (1.44 to 2.01) |  |  |
|  | Cough | 1.31 (1.07 to 1.61) |  |  |
|  | Increased respiratory rate | 1.42 (1.19 to 1.69) |  |  |
|  | Nasal flaring | 0.69 (0.56 to 0.87) |  |  |
|  | Auscultatory crackles | 1.41 (1.16 to 1.71) |  |  |
|  | Oral candidiasis | 2.72 (2.03 to 3.64) |  |  |
|  | Edema | 1.83 (1.67 to 2.44) |  |  |
|  | Depigmented/reddish hair | 2.08 (1.64 to 2.64) |  |  |
|  | Swollen lymph nodes | 1.87 (1.41 to 2.49) |  |  |
|  | Ear discharge | 1.74 (0.59 to 1.16) |  |  |
|  | Prostration | 1.44 (1.17 to 1.77) |  |  |
|  | Malaria Positive | 0.43 (0.35 to 0.52) |  |  |
|  | Malaria Test not done | 0.84 (0.68 to 1.04) |  |  |
|  | HIV Positive | 1.80 (1.07 to 3.01) |  |  |
|  | HIV Negative | 0.53 (0.35 to 0.80) |  |  |
|  | Outcome of Admission: Absconded | 5.50 (4.45 to 6.79) |  |  |
|  | Outcome of Admission: Transferred | 4.57 (3.36 to 6.21) |  |  |
|  | | | | |
| Madrid L, et al. *Pediatrics*. 2019. (Model 2, 90-day outcome) | Age 4m-<1 y | 0.93* (0.72 to 1.20) | 0.78 (0.75 to 0.82) | No Published External Validation Studies |
|  | Age 1-5 y | 0.71 (0.54 to 0.92) |  |  |
|  | Age >5 y | 0.54 (0.38 to 0.76) |  |  |
|  | Rainy season | 1.22 (1.04 to 1.44) |  |  |
|  | WHZ z score >−2 to <−1 | 1.27 (0.77 to 2.07) |  |  |
|  | WHZ z score >−3 to <−2 | 2.44 (1.51 to 3.93) |  |  |
|  | WHZ z score <−3 | 3.28 (2.08 to 5.16) |  |  |
|  | WHZ z score Unknown | 3.09 (2.19 to 4.35) |  |  |
|  | Diarrhea | 1.70 (1.44 to 2.01) |  |  |
|  | Cough | 1.31 (1.07 to 1.61) |  |  |
|  | Increased respiratory rate | 1.42 (1.19 to 1.69) |  |  |
|  | Nasal flaring | 0.69 (0.56 to 0.87) |  |  |
|  | Auscultatory crackles | 1.41 (1.16 to 1.71) |  |  |
|  | Oral candidiasis | 2.72 (2.03 to 3.64) |  |  |
|  | Edema | 1.83 (1.67 to 2.44) |  |  |
|  | Depigmented/reddish hair | 2.08 (1.64 to 2.64) |  |  |
|  | Swollen lymph nodes | 1.87 (1.41 to 2.49) |  |  |
|  | Ear discharge | 1.74 (0.59 to 1.16) |  |  |
|  | Prostration | 1.44 (1.17 to 1.77) |  |  |
|  | Malaria Positive | 0.43 (0.35 to 0.52) |  |  |
|  | Malaria Test not done | 0.84 (0.68 to 1.04) |  |  |
|  | HIV Positive | 1.80 (1.07 to 3.01) |  |  |
|  | HIV Negative | 0.53 (0.35 to 0.80) |  |  |
|  | Outcome of Admission: Absconded | 5.50 (4.45 to 6.79) |  |  |
|  | Outcome of Admission: Transferred | 4.57 (3.36 to 6.21) |  |  |
|  | | | | |
| Madrid L, et al. *Pediatrics*. 2019. (Model 3, 30-day outcome) | Age 4m-<1 y | 0.79* (0.62 to 1.03) | 0.78 (0.72 to 0.82) | No Published External Validation Studies |
|  | Age 1-5 y | 0.66 (0.51 to 0.86) |  |  |
|  | Age >5 y | 0.55 (0.39 to 0.77) |  |  |
|  | Rainy season | 1.25 (1.07 to 1.46) |  |  |
|  | WHZ z score >−2 to <−1 | 1.32 (0.81 to 2.14) |  |  |
|  | WHZ z score >−3 to <−2 | 2.30 (1.41 to 3.75) |  |  |
|  | WHZ z score <−3 | 4.16 (2.71 to 6.40) |  |  |
|  | WHZ z score Unknown | 3.72 (2.64 to 5.23) |  |  |
|  | Diarrhea | 1.58 (1.32 to 1.89) |  |  |
|  | Cough | 1.25 (1.02 to 1.53) |  |  |
|  | Breathing difficulties | 1.36 (1.09 to 1.70) |  |  |
|  | Increased respiratory rate | 1.27 (1.07 to 1.52) |  |  |
|  | Skin pinch goes back slowly | 1.51 (1.20 to 1.90) |  |  |
|  | Nasal flaring | 0.79 (0.65 to 0.97) |  |  |
|  | Auscultatory crackles | 1.44 (1.19 to 1.75) |  |  |
|  | Oral candidiasis | 3.51 (2.70 to 4.58) |  |  |
|  | Edema | 2.48 (1.88 to 3.27) |  |  |
|  | Depigmented/reddish hair | 2.42 (1.90 to 3.07) |  |  |
|  | Swollen lymph nodes | 2.23 (1.70 to 2.93) |  |  |
|  | Ear discharge | 1.76 (1.24 to 2.49) |  |  |
|  | Prostration | 1.41 (1.15 to 1.73) |  |  |
|  | | | | |
| Madrid L, et al. *Pediatrics*. 2019. (Model 3, 60-day outcome) | Age 4m-<1 y | 0.79* (0.62 to 1.03) | 0.75 (0.71 to 0.79) | No Published External Validation Studies |
|  | Age 1-5 y | 0.66 (0.51 to 0.86) |  |  |
|  | Age >5 y | 0.55 (0.39 to 0.77) |  |  |
|  | Rainy season | 1.25 (1.07 to 1.46) |  |  |
|  | WHZ z score >−2 to <−1 | 1.32 (0.81 to 2.14) |  |  |
|  | WHZ z score >−3 to <−2 | 2.30 (1.41 to 3.75) |  |  |
|  | WHZ z score <−3 | 4.16 (2.71 to 6.40) |  |  |
|  | WHZ z score Unknown | 3.72 (2.64 to 5.23) |  |  |
|  | Diarrhea | 1.58 (1.32 to 1.89) |  |  |
|  | Cough | 1.25 (1.02 to 1.53) |  |  |
|  | Breathing difficulties | 1.36 (1.09 to 1.70) |  |  |
|  | Increased respiratory rate | 1.27 (1.07 to 1.52) |  |  |
|  | Skin pinch goes back slowly | 1.51 (1.20 to 1.90) |  |  |
|  | Nasal flaring | 0.79 (0.65 to 0.97) |  |  |
|  | Auscultatory crackles | 1.44 (1.19 to 1.75) |  |  |
|  | Oral candidiasis | 3.51 (2.70 to 4.58) |  |  |
|  | Edema | 2.48 (1.88 to 3.27) |  |  |
|  | Depigmented/reddish hair | 2.42 (1.90 to 3.07) |  |  |
|  | Swollen lymph nodes | 2.23 (1.70 to 2.93) |  |  |
|  | Ear discharge | 1.76 (1.24 to 2.49) |  |  |
|  | Prostration | 1.41 (1.15 to 1.73) |  |  |
|  | | | | |
| Madrid L, et al. *Pediatrics*. 2019. (Model 3, 90-day outcome) | Age 4m-<1 y | 0.79* (0.62 to 1.03) | 0.75 (0.71 to 0.78) | No Published External Validation Studies |
|  | Age 1-5 y | 0.66 (0.51 to 0.86) |  |  |
|  | Age >5 y | 0.55 (0.39 to 0.77) |  |  |
|  | Rainy season | 1.25 (1.07 to 1.46) |  |  |
|  | WHZ z score >−2 to <−1 | 1.32 (0.81 to 2.14) |  |  |
|  | WHZ z score >−3 to <−2 | 2.30 (1.41 to 3.75) |  |  |
|  | WHZ z score <−3 | 4.16 (2.71 to 6.40) |  |  |
|  | WHZ z score Unknown | 3.72 (2.64 to 5.23) |  |  |
|  | Diarrhea | 1.58 (1.32 to 1.89) |  |  |
|  | Cough | 1.25 (1.02 to 1.53) |  |  |
|  | Breathing difficulties | 1.36 (1.09 to 1.70) |  |  |
|  | Increased respiratory rate | 1.27 (1.07 to 1.52) |  |  |
|  | Skin pinch goes back slowly | 1.51 (1.20 to 1.90) |  |  |
|  | Nasal flaring | 0.79 (0.65 to 0.97) |  |  |
|  | Auscultatory crackles | 1.44 (1.19 to 1.75) |  |  |
|  | Oral candidiasis | 3.51 (2.70 to 4.58) |  |  |
|  | Edema | 2.48 (1.88 to 3.27) |  |  |
|  | Depigmented/reddish hair | 2.42 (1.90 to 3.07) |  |  |
|  | Swollen lymph nodes | 2.23 (1.70 to 2.93) |  |  |
|  | Ear discharge | 1.76 (1.24 to 2.49) |  |  |
|  | Prostration | 1.41 (1.15 to 1.73) |  |  |
