# Supplementary material for: Systematic review of risk assessment tools for post-discharge mortality among children in sub-Saharan Africa
Source: PLOS Glob Public Health. 2025 Jul 1;5(7):e0004788. doi: 10.1371/journal.pgph.0004788 (PMC12212496; doi:10.1371/journal.pgph.0004788)
Supplement: S2 Table — (DOCX) [file pgph.0004788.s002.docx]

**S2 Table.** Bias assessment for risk assessment tools for post-discharge mortality prediction among children in sub-Saharan Africa according to the Prediction Model Risk of Bias Assessment Tool (PROBAST)

| Study | Risk of Bias (ROB) | | | | Applicability | | | Overall | |
| --- | --- | --- | --- | --- | --- | --- | --- | --- | --- |
|  | Participants | Predictors | Outcome | Analysis | Participants | Predictors | Outcome | ROB | Applicability |
| Wiens MO, et al. *BMJ Open*. 2015. | + | + | + | ? | + | + | + | ? | + |
| Madrid L, et al. *Pediatrics*. 2019. | ? | + | ? | ? | + | + | + | ? | ? |
| Talbert A, et al. *BMC Med*. 2019. | + | + | ? | - | + | + | ? | ? | + |
| Ahmed SM, et al. *PLOS Glob Public Health*. 2023. | + | + | + | + | + | + | + | + | + |
| Rees CA, et al. *J Pediatrics*. 2024. | ? | + | + | + | + | + | + | ? | + |
| Rees CA, et al. *BMJ Open*. 2024. | + | + | + | - | + | + | + | ? | + |
| Wiens MO, et al. *PLOS Glob Public Health*. 2024. | + | + | + | ? | + | + | + | ? | + |

* + indicates low risk of bias/low concern regarding applicability; − indicates high risk of bias/high concern regarding applicability; and ? indicates unclear risk of bias/unclear concern regarding applicability.
