# Supplementary material for: Systematic review of risk assessment tools for post-discharge mortality among children in sub-Saharan Africa
Source: PLOS Glob Public Health. 2025 Jul 1;5(7):e0004788. doi: 10.1371/journal.pgph.0004788 (PMC12212496; doi:10.1371/journal.pgph.0004788)
Supplement: S1 Appendix — (DOCX) [file pgph.0004788.s003.docx]

**S1 Appendix**. Search terms used to identify publications reporting on risk assessment tools for post-discharge mortality among children in sub-Saharan Africa

**PubMed 1857 07/31/2024**
(“post-discharge mortal*”[tw] OR “post discharge mortal*”[tw] OR PDM) OR (("Hospitalization"[Mesh] OR hospital*[tw]) AND ("Mortality"[Mesh] OR mortal*[tw] OR death*[tw] OR fatal*[tw]) AND ("Follow-Up Studies"[Mesh] OR "Longitudinal Studies"[Mesh] OR followup[tw] or “follow up”[tw] OR “follow-up”[tw] OR postdischarge[tw] OR post-discharge[tw] OR “post discharge”[tw] OR “after discharge”[tw] OR “after-discharge”[tw] OR “after hospital*”[tw] OR “after-hospital”[tw] OR “post hospital*”[tw] OR “post-hospital*”[tw] OR “patient discharge”[tw]))

("Adolescent"[Mesh] OR adolesc*[tw] OR baby[tw] OR babies[tw] OR boy[tw] OR boys[tw] OR child*[tw] OR "Child"[Mesh] OR girl*[tw] OR Infan*[tw] OR "Infant"[Mesh] OR juvenil*[tw] OR minors[tw] OR neonat*[tw] OR neo-nat*[tw] OR newborn*[tw] OR new-born*[tw] OR pediat*[tw] OR paediat*[tw] OR "Pediatrics"[Mesh] OR "Pediatricians"[Mesh] OR perinat*[tw] OR pre-adolescen*[tw] OR preadolescen*[tw] OR prematur*[tw] OR prepubesc*[tw] OR pre-pubesc*[tw] OR pubescen*[tw] OR "school age*"[tw] OR schoolchild*[tw] OR "school child*"[tw] OR teen*[tw] OR toddler*[tw] OR under*age*[tw] OR young[tw] OR "young adult"[MeSH Terms] OR youth*[tw] OR preschool*[tw] OR "pre-school"[tw] OR kindergarden*[tw] OR kindergarten*[tw] OR "elementary school*"[tw] OR "middle school*"[tw] OR "junior high*"[tw] OR "primary school*"[tw] OR "secondary school*"[tw] OR "high school*"[tw])

(Angola[tw] OR  Benin[tw] OR Botswana[tw] OR "Burkina Faso"[tw] OR Burundi[tw] OR "Cabo Verde"[tw] OR "Cape Verde"[tw] OR Cameroon[tw] OR “Central African Republic”[tw] OR Chad[tw] OR Comoros[tw] OR Congo[tw] OR "Cote d'Ivoire"[tw] OR "Ivory Coast"[tw] OR Djibouti [tw] OR "Equatorial Guinea" [tw] OR Eritrea [tw] OR Eswatini [tw] OR Ethiopia [tw] OR Gabon [tw] OR Gambia [tw] OR Ghana [tw] OR Guinea [tw] OR "Guinea-Bissau" [tw] OR Kenya [tw] OR Lesotho [tw] OR Liberia[tw] OR Madagascar [tw] OR Malawi [tw] OR Mali [tw] OR Mauritania [tw] OR Mauritius [tw] OR Mozambique [tw] OR Namibia [tw] OR Niger [tw] OR Nigeria [tw] OR Rwanda [tw] OR "Sao Tome"[tw] OR  Principe [tw] OR Senegal[tw] OR Seychelles [tw] OR "Sierra Leone"[tw] OR Somalia [tw] OR "South Africa"[tw] OR  "Sub Saharan"[tw]  OR “sub-sahara*”[tw] OR Sudan [tw] OR Tanzania[tw] OR Togo[tw] OR Uganda[tw] OR Zambia[tw] OR Zimbabwe [tw] OR “southern africa*”[tw] OR “central africa*”[tw] OR “eastern africa*”[tw] OR “east africa*”[tw] OR “western africa*”[tw] OR “west africa*”[tw])

(#1 AND #2 AND #3) NOT ("animals"[MeSH Terms] NOT "humans"[MeSH Terms])

**Web of Science 1101 07/31/2024**

(“post-discharge mortal*” OR “post discharge mortal*” OR PDM) OR (hospital* AND (mortal* OR death* OR fatal*) AND (followup or “follow up” OR “follow-up” OR postdischarge OR post-discharge OR “post discharge” OR “after discharge” OR “after-discharge” OR “after hospital*” OR “after-hospital” OR “post hospital*” OR “post-hospital*” OR “patient discharge”))

(adolesc* OR baby OR babies OR boy OR boys OR child* OR girl* OR Infan* OR juvenil* OR minors OR neonat* OR neo-nat* OR newborn* OR new-born* OR pediat* OR paediat* OR perinat* OR pre-adolescen* OR preadolescen* OR prematur* OR prepubesc* OR pre-pubesc* OR pubescen* OR "school age*" OR schoolchild* OR "school child*" OR teen* OR toddler* OR under*age* OR young OR youth* OR preschool* OR "pre-school" OR kindergarden* OR kindergarten* OR "elementary school*" OR "middle school*" OR "junior high*" OR "primary school*" OR "secondary school*" OR "high school*")

(Angola OR  Benin OR Botswana OR "Burkina Faso" OR Burundi OR "Cabo Verde" OR "Cape Verde" OR Cameroon OR “Central African Republic” OR Chad OR Comoros OR Congo OR "Cote d'Ivoire" OR "Ivory Coast" OR Djibouti OR "Equatorial Guinea" OR Eritrea  OR Eswatini  OR Ethiopia  OR Gabon  OR Gambia  OR Ghana  OR Guinea  OR "Guinea-Bissau"  OR Kenya  OR Lesotho  OR Liberia OR Madagascar  OR Malawi  OR Mali OR Mauritania  OR Mauritius  OR Mozambique  OR Namibia  OR Niger  OR Nigeria  OR Rwanda  OR "Sao Tome" OR  Principe  OR Senegal OR Seychelles  OR "Sierra Leone" OR Somalia  OR "South Africa" OR  "Sub Saharan"  OR “sub-sahara*” OR Sudan  OR Tanzania OR Togo OR Uganda OR Zambia OR Zimbabwe OR “southern africa*” OR “central africa*” OR “eastern africa*” OR “east africa*” OR “western africa*” OR “west africa*”

#1 AND #2 AND #3)

**EMBASE 3469 07/31/2024**

(“post-discharge mortal*” OR “post discharge mortal*” OR PDM)

(hospital* AND (mortal* OR death* OR fatal*) AND (followup or “follow up” OR “follow-up” OR postdischarge OR post-discharge OR “post discharge” OR “after discharge” OR “after-discharge” OR “after hospital*” OR “after-hospital” OR “post hospital*” OR “post-hospital*” OR “patient discharge”))

(adolesc* OR baby OR babies OR boy OR boys OR child* OR girl* OR Infan* OR juvenil* OR minors OR neonat* OR neo-nat* OR newborn* OR new-born* OR pediat* OR paediat* OR perinat* OR pre-adolescen* OR preadolescen* OR prematur* OR prepubesc* OR pre-pubesc* OR pubescen* OR "school age*" OR schoolchild* OR "school child*" OR teen* OR toddler* OR under*age* OR young OR youth* OR preschool* OR "pre-school" OR kindergarden* OR kindergarten* OR "elementary school*" OR "middle school*" OR "junior high*" OR "primary school*" OR "secondary school*" OR "high school*")

(Angola OR  Benin OR Botswana OR "Burkina Faso" OR Burundi OR "Cabo Verde" OR "Cape Verde" OR Cameroon OR “Central African Republic” OR Chad OR Comoros OR Congo OR "Ivory Coast" OR Djibouti OR "Equatorial Guinea" OR Eritrea  OR Eswatini  OR Ethiopia  OR Gabon  OR Gambia  OR Ghana  OR Guinea  OR "Guinea-Bissau"  OR Kenya  OR Lesotho  OR Liberia OR Madagascar  OR Malawi  OR Mali OR Mauritania  OR Mauritius  OR Mozambique  OR Namibia  OR Niger  OR Nigeria  OR Rwanda  OR "Sao Tome" OR  Principe  OR Senegal OR Seychelles  OR "Sierra Leone" OR Somalia  OR "South Africa" OR  "Sub Saharan"  OR “sub-sahara*” OR Sudan  OR Tanzania OR Togo OR Uganda OR Zambia OR Zimbabwe OR “southern africa*” OR “central africa*” OR “eastern africa*” OR “east africa*” OR “western africa*” OR “west africa*”)

(#1 OR #2) AND #3 AND #4

Limits: humans, embase, article/it, conference paper/it, review/it

**Cochrane Reviews 15 Cochrane Trials 507 07/31/2024**

(“post-discharge mortal*” OR “post discharge mortal*” OR PDM) OR (hospital* AND (mortal* OR death* OR fatal*) AND (followup or “follow up” OR “follow-up” OR postdischarge OR post-discharge OR “post discharge” OR “after discharge” OR “after-discharge” OR “after hospital*” OR “after-hospital” OR “post hospital*” OR “post-hospital*” OR “patient discharge”))

(adolesc* OR baby OR babies OR boy OR boys OR child* OR girl* OR Infan* OR juvenil* OR minors OR neonat* OR neo-nat* OR newborn* OR new-born* OR pediat* OR paediat* OR perinat* OR pre-adolescen* OR preadolescen* OR prematur* OR prepubesc* OR pre-pubesc* OR pubescen* OR "school age*" OR schoolchild* OR "school child*" OR teen* OR toddler* OR under*age* OR young OR youth* OR preschool* OR "pre-school" OR kindergarden* OR kindergarten* OR "elementary school*" OR "middle school*" OR "junior high*" OR "primary school*" OR "secondary school*" OR "high school*")

(Angola OR  Benin OR Botswana OR "Burkina Faso" OR Burundi OR "Cabo Verde" OR "Cape Verde" OR Cameroon OR “Central African Republic” OR Chad OR Comoros OR Congo OR "Cote d'Ivoire" OR "Ivory Coast" OR Djibouti OR "Equatorial Guinea" OR Eritrea  OR Eswatini  OR Ethiopia  OR Gabon  OR Gambia  OR Ghana  OR Guinea  OR "Guinea-Bissau"  OR Kenya  OR Lesotho  OR Liberia OR Madagascar  OR Malawi  OR Mali OR Mauritania  OR Mauritius  OR Mozambique  OR Namibia  OR Niger  OR Nigeria  OR Rwanda  OR "Sao Tome" OR  Principe  OR Senegal OR Seychelles  OR "Sierra Leone" OR Somalia  OR "South Africa" OR  "Sub Saharan"  OR “sub-saharan” OR Sudan  OR Tanzania OR Togo OR Uganda OR Zambia OR Zimbabwe OR “southern africa” OR “central africa” OR “eastern africa” OR “east africa” OR “western africa” OR “west africa”)

#1 AND #2 AND #3

**CAB Global Health 5 07/31/2024**

(“post-discharge mortal*” OR “post discharge mortal*” OR PDM) OR (hospital* AND (mortal* OR death* OR fatal*) AND (followup or “follow up” OR “follow-up” OR postdischarge OR post-discharge OR “post discharge” OR “after discharge” OR “after-discharge” OR “after hospital*” OR “after-hospital” OR “post hospital*” OR “post-hospital*” OR “patient discharge”))

(adolesc* OR baby OR babies OR boy OR boys OR child* OR girl* OR Infan* OR juvenil* OR minors OR neonat* OR neo-nat* OR newborn* OR new-born* OR pediat* OR paediat* OR perinat* OR pre-adolescen* OR preadolescen* OR prematur* OR prepubesc* OR pre-pubesc* OR pubescen* OR "school age*" OR schoolchild* OR "school child*" OR teen* OR toddler* OR under*age* OR young OR youth* OR preschool* OR "pre-school" OR kindergarden* OR kindergarten* OR "elementary school*" OR "middle school*" OR "junior high*" OR "primary school*" OR "secondary school*" OR "high school*")

(Angola OR  Benin OR Botswana OR "Burkina Faso" OR Burundi OR "Cabo Verde" OR "Cape Verde" OR Cameroon OR “Central African Republic” OR Chad OR Comoros OR Congo OR "Cote d'Ivoire" OR "Ivory Coast" OR Djibouti OR "Equatorial Guinea" OR Eritrea  OR Eswatini  OR Ethiopia  OR Gabon  OR Gambia  OR Ghana  OR Guinea  OR "Guinea-Bissau"  OR Kenya  OR Lesotho  OR Liberia OR Madagascar  OR Malawi  OR Mali OR Mauritania  OR Mauritius  OR Mozambique  OR Namibia  OR Niger  OR Nigeria  OR Rwanda  OR "Sao Tome" OR  Principe  OR Senegal OR Seychelles  OR "Sierra Leone" OR Somalia  OR "South Africa" OR  "Sub Saharan"  OR “sub-sahara*” OR Sudan  OR Tanzania OR Togo OR Uganda OR Zambia OR Zimbabwe OR “southern africa*” OR “central africa*” OR “eastern africa*” OR “east africa*” OR “western africa*” OR “west africa*”)

#1 AND #2 AND #3

**ProQuest Disserations and Theses 65 07/31/2024**

noft((“post-discharge mortal*” OR “post discharge mortal*” OR PDM) OR (hospital* AND (mortal* OR death* OR fatal*) AND (followup or “follow up” OR “follow-up” OR postdischarge OR post-discharge OR “post discharge” OR “after discharge” OR “after-discharge” OR “after hospital*” OR “after-hospital” OR “post hospital*” OR “post-hospital*” OR “patient discharge”))) AND noft(adolesc* OR baby OR babies OR boy OR boys OR child* OR girl* OR Infan* OR juvenil* OR minors OR neonat* OR neo-nat* OR newborn* OR new-born* OR pediat* OR paediat* OR perinat* OR pre-adolescen* OR preadolescen* OR prematur* OR prepubesc* OR pre-pubesc* OR pubescen* OR "school age*" OR schoolchild* OR "school child*" OR teen* OR toddler* OR under*age* OR young OR youth* OR preschool* OR "pre-school" OR kindergarden* OR kindergarten* OR "elementary school*" OR "middle school*" OR "junior high*" OR "primary school*" OR "secondary school*" OR "high school*") AND noft(Angola OR Benin OR Botswana OR "Burkina Faso" OR Burundi OR "Cabo Verde" OR "Cape Verde" OR Cameroon OR “Central African Republic” OR Chad OR Comoros OR Congo OR "Cote d'Ivoire" OR "Ivory Coast" OR Djibouti OR "Equatorial Guinea" OR Eritrea OR Eswatini OR Ethiopia OR Gabon OR Gambia OR Ghana OR Guinea OR "Guinea-Bissau" OR Kenya OR Lesotho OR Liberia OR Madagascar OR Malawi OR Mali OR Mauritania OR Mauritius OR Mozambique OR Namibia OR Niger OR Nigeria OR Rwanda OR "Sao Tome" OR Principe OR Senegal OR Seychelles OR "Sierra Leone" OR Somalia OR "South Africa" OR "Sub Saharan" OR “sub-saharan” OR Sudan OR Tanzania OR Togo OR Uganda OR Zambia OR Zimbabwe OR “southern africa” OR “central africa” OR “eastern africa” OR “east africa” OR “western africa” OR “west africa” )
